# Using digital phenotyping to capture depression symptom variability: detecting naturalistic variability in depression symptoms across one year using passively collected wearable movement and sleep data

**DOI:** 10.1038/s41398-023-02669-y

**Published:** 2023-12-09

**Authors:** George D. Price, Michael V. Heinz, Seo Ho Song, Matthew D. Nemesure, Nicholas C. Jacobson

**Affiliations:** 1 Center for Technology and Behavioral Health, Geisel School of Medicine, Dartmouth College, Lebanon, NH USA; 2 Quantitative Biomedical Sciences Program, Dartmouth College, Lebanon, NH USA; 3 Department of Psychiatry, Geisel School of Medicine, Dartmouth College, Lebanon, NH USA; 4 Department of Psychiatry, Beth Israel Deaconess Medical Center, Boston, MA USA; 5 Digital Data Design Institute, Harvard Business School, Harvard University, Cambridge, MA USA; 6 Department of Biomedical Data Science, Geisel School of Medicine, Dartmouth College, Lebanon, NH USA

**Keywords:** Depression, Predictive markers

## Abstract

Major Depressive Disorder (MDD) presents considerable challenges to diagnosis and management due to symptom variability across time. Only recent work has highlighted the clinical implications for interrogating depression symptom variability. Thus, the present work investigates how sociodemographic, comorbidity, movement, and sleep data is associated with long-term depression symptom variability. Participant information included (*N* = 939) baseline sociodemographic and comorbidity data, longitudinal, passively collected wearable data, and Patient Health Questionnaire-9 (PHQ-9) scores collected over 12 months. An ensemble machine learning approach was used to detect long-term depression symptom variability via: (i) a domain-driven feature selection approach and (ii) an exhaustive feature-inclusion approach. SHapley Additive exPlanations (SHAP) were used to interrogate variable importance and directionality. The composite domain-driven and exhaustive inclusion models were both capable of moderately detecting long-term depression symptom variability (*r* = 0.33 and *r* = 0.39, respectively). Our results indicate the incremental predictive validity of sociodemographic, comorbidity, and passively collected wearable movement and sleep data in detecting long-term depression symptom variability.

## Introduction

Major Depressive Disorder (MDD) is highly prevalent and burdensome, socially and economically. An estimated 8% of all U.S. adults (~21 M) experienced a depressive episode in the last year [1], and an estimated 6% (15 M) experienced associated severe functional impairment [1]. Depression is ranked in the top twenty leading causes of disability, globally [2] and is estimated to cost $326 billion USD annually, an increase of 38% in the last decade [3]. Many people with MDD do not receive treatment, with one in three people with active symptoms failing to receive care [1]. Further, MDD is frequently misdiagnosed by primary care, which is often the first point of contact for those with clinical symptoms [4].

MDD presents considerable challenges to effective diagnosis and management, due, in part, to its dynamic nature and variable trajectory [5]. The longitudinal course of MDD, as described by the DSM-5, allows for considerable variability across persons, such that some individuals may experience only discrete episodes separated by long periods of remission, while others experience chronic, unrelenting symptoms over years [6]. Research to date has explored person-to-person differences in depression course and variability over time, with empirical evidence for heterogeneity in symptom trajectory [7–9], as well as difficulty in predicting longitudinal course [10]. These findings suggest that cross-sectional severity (“level of depression”) and presence (“depressed vs. not depressed”) outcomes alone, while providing informative “snapshots” in time, are insufficient for understanding the naturalistic course of MDD, and thus, the core nature of MDD.

We posit that depression symptom variability, per se, is an important outcome, which has meaningful basic science and translational implications. For the purpose of our study, we define depression symptom variability to mean the degree of within-person variation in reported depression symptom severity across time. Indeed, research to date examining depression temporal dynamics (Nemesure et al., [11]), has revealed considerable within and between-person symptom variability over time. We provide a theoretical and empirical basis for the importance of depression symptom variability as an outcome. First, variability is important to explore as a core metric of depression’s naturalistic, longitudinal course. Together with other summative longitudinal metrics, such as mean severity, variability provides an important summary of depression’s longitudinal course. Depression symptom variability is a necessary precondition for relapse and remission (i.e., major depressive episodes), which are important outcome and prognostic markers in MDD [6]. Further, depression temporal variability may help to inform diagnostic distinctions, such as that between MDD and Persistent Depressive Disorder (PDD), with the latter theoretically showing less long-term temporal variability than the former as well as more severe functional impairment [12]. Therefore, a nuanced understanding of depression’s course, including an understanding of those factors associated with symptom variability, is fundamental to effective assessment and management. A highly variable course, for instance, would require more frequent assessments to accurately describe the disorder trajectory, and likely more temporally dynamic interventions.

Second, depression symptom variability has been associated with important clinical, prognostic, and treatment outcomes. Specifically, higher depression symptom variability has been positively associated with (i) higher risk of suicide attempts [13], (ii) lower family functioning (in maternal depression) [14], (iii) cognitive decline [15], and (iv) pathological narcissism [16] (an important prognostic marker for mental health treatment) [17]. Depressed mood variability has also been shown to interact with perceived self-esteem instability in predicting future depression at six-month follow-up [18], and a variable, chronic depression course has been associated with all-cause mortality in older adults [19]. In addition, rapid symptom fluctuation in depressed people has been associated with involvement in violence [20]. Given these impactful clinical and prognostic associations, it is of considerable importance to understand naturalistic depression symptom variability, including the personalized features which may contribute to a fluctuating course.

Of important transdiagnostic consideration, there is face validity that depression variability may have a relation to affective instability, the latter of which has been studied in relation to depression utilizing repeat assessment of both high and low-arousal negative affect features [21]; low-arousal negative affect features (e.g., “tired”, “bored”, “droopy”) [22] have considerable overlap with the core neurovegetative depressive symptoms including low energy, depressed mood, and reduced interest [6]. Thus it may be a reasonable assumption that affective instability may be at least partially explained by temporal depression variability, and therefore understanding depression variability may help in understanding affective instability, which is also an important consideration in borderline personality and bipolar disorders [23].

Machine learning methods, operating on highly dimensional datasets, have shown great promise in modeling important clinically relevant outcomes in MDD [24–26]. Advances in computing power and passive data streaming have made possible the application of ecologically valid, person-generated health data (e.g., sleep, movement) to personalized depression models [24], complementing more traditional demographic features. Price et al., for example, utilized actigraphy data to effectively detect MDD presence in a large cohort [27]. Naturalistic movement and sleep data are promising candidates for modeling MDD symptom variability, given their established relationship to major depressive episodes and their capacity for predicting depression severity [28, 29]. In particular, sleep and movement problems are core features of depression [6], and sleep problems are a known risk factor for depression recurrence [30], a plausible driver of long-term symptom variability. In addition, such passively collected features have contributed to empirical support for MDD-associated (1) sleep and circadian rhythm irregularities [31, 32], (2) reduced locomotion [33], and (3) reduced daily activity [34]. These efforts inform our understanding of features associated with depression presence and severity, and thereby serve as a benchmark for identifying biodemographic and behavioral characteristics that may also have an association with long-term depression symptom variability.

To build upon efforts by Makhmutova et al. in the development of the Prediction of Severity-Change Depression (PSYCHE-D) model and data source [35, 36], the present work leveraged a stacked ensemble machine learning approach applied to baseline biodemographic (i.e., sociodemographic and comorbidity) features and objective, wearable passively collected movement and sleep data, to explore factors associated with long-term depression symptom variability. Methodologically, our work is unique in our direct model comparisons on the basis of feature selection and feature-type. First, we compared a model trained on theory-informed feature selection against a parallel model trained on an exhaustive feature set. Second, we compare a model trained on baseline demographic features to a parallel model trained on passively derived sleep and activity features. Further, we examine the incremental predictive gain when combining both types of features; for all models we utilize a robust stacked ensemble approach. We hypothesized that (1) features having known association with depression presence and severity would also associate with long-term symptom variability. Further, (2) we hypothesized that biodemographic and objective passively collected movement and sleep data each contain complementary information and, thus, when combined would produce improved model prediction compared to either singular information modality, as accounting for complementarity during feature selection has been shown to increase model performance [37, 38]. To test our hypotheses, we used 12-month longitudinal data [39] comprising personal biodemographic data, movement, and sleep metrics statistically derived from passively collected wearable accelerometry data, and quarterly PHQ-9 scores. A cross-validation framework, coupled with a stacked ensemble machine learning approach, was implemented to model depression symptom variability using features with empirical associations with depression. For model interpretability, we used an algorithmic approach to quantify the relative importance and directionality of biodemographic features, statistical movement, and sleep features, and both in concert for predicting depression symptom variability.

## Methods

### Study sample

**Fig. 1:**
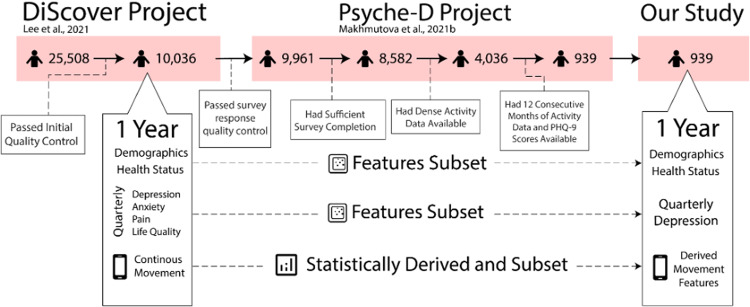
A flow diagram representing the selection and exclusion of participants, which led to the 939-participant sample in the present work. From top to bottom: Headers at the top of the diagram reflect projects, with citations, from which the data originated. Below the headers, we show the absolute numbers of participants, changing with further exclusion, during each stage of the project. Dialogue bubbles provide detail at a stage where participants were excluded. Large rectangular dialogue boxes contain high-level detail regarding features included at each stage. Gradient arrows indicate feature change or subsetting that occurred to produce the feature set used in the present work.

The present work used publicly available biodemographic, wearable passively collected movement and sleep, and depression symptom data originally collected over a 12-month period provided in the PSYCHE-D dataset [40], which was captured as part of the DiSCover Project developed by Evidation Health [39]. Participants were originally recruited via Achievement, a community of adults in the United States that can connect consumer-grade fitness applications and wearable (e.g., Fitbit, Garmin) to the study platform. Participant inclusion was limited in the present analyses to individuals with twelve consecutive months of objective accelerometer information, reflecting non-missing values for some or all of the related movement and sleep metrics for each month, and a reported Patient Health Questionnaire-9 (PHQ-9) [41] composite score completed at baseline and every subsequent 3-month time point for the 12-month study period (*N* = 939, 70.61% female, 29.39% male, age_*mean*_ = 42.55 ± 10.23, 91.37% White, 4.69% Black, 4.05% Hispanic, 2.66% Asian, 2.23% Race not specified, 10.81% required financial assistance from the government) (see Fig. 1). A full description of the original DiSCover Project study design, recruitment protocols, and participant baseline demographic information is provided by Lee et al. [39].

### Study measures

The original PSYCHE-D dataset contains 150 person-generated health data (PGHD) features reflecting baseline biodemographic information, derived passively collected movement and sleep information, and Patient Health Questionnaire-9 (PHQ-9) composite scores (PHQ-9_*mean*_ = 6.80 ± 5.72; 42.79% No Depressive Symptoms, 28.78% Mild Depressive Symptoms, 17.61% Moderate Depressive Symptoms, 7.41% Moderately Severe Depressive Symptoms, 3.41% Severe Depressive Symptoms) [40]; a common screening tool for MDD [42] consisting of nine items which reflect the degree to which each item was bothersome over the last two weeks (e.g., feeling down, depressed, or hopeless) [41]. Makhmutova et al. describe the PGHD feature collection and processing in further detail [35]. The dataset was subset for the present analyses to 20 features consisting of a combination of 8 baseline biodemographic (i.e., Sex, Race, BMI, Pregnancy Status, Money Assistance, Comorbid Diabetes Type I, Comorbid Diabetes Type II, Comorbid Migraines), and 12 derived passively collected movement and sleep data (i.e., Average Awake Activity, Low Physical Activity Duration, Moderate-to-Vigorous Activity Duration, Active Day Count, Sedentary Day Count, Nighttime Sleep Variability, Average Weekday Sleep, Average Weekend Sleep, Sleep Start Time, Variability In Sleep Start Time, Weekly Hypersomnia Count, Weekly Hyposomnia Count). These features were chosen based on known direct or indirect associations with depression, outlined in Supplementary Table 1, as feature engineering and selection informed by domain knowledge has been shown to improve predictive performance and model interpretability [43].

### Data preprocessing

All data preprocessing was performed in R (v 4.0.2) [44]. Baseline biodemographic feature data types were interrogated and converted according to their reporting structure (e.g., Migraine comorbidity was converted from numerical to categorical). To account for the missingness of certain biodemographic and movement and sleep-related metrics, multivariate imputation by chained equations (mice) with predictive mean matching was implemented using the *mice* package in R [45], as mice is well-suited to handling high proportions of missing data, and captures the uncertainty associated with approximating missing information [46]. Across all participants, 0.08% of the subsetted biodemographic information was missing, and 15.64% of the subsetted passively collected movement and sleep-related metrics information was missing. Resultantly, five imputed datasets were generated, reflecting the plausible distribution of missing information, and used for subsequent analyses. Following imputation, summative metrics of the longitudinal passive-collected movement and sleep features were derived to represent the average and variability of each selected feature across the twelve-month data collection period. The average was calculated as the mean of the feature’s values, and variability was calculated as the root mean square of successive differences (RMSSD) of the respective feature. The summative features were derived to reflect longitudinal movement and sleep behaviors, as well as avoid a nested data structure, such that each participant could be represented as a single row with their fixed baseline biodemographic features and their statistically derived movement and sleep features. To interrogate the naturalistic fluctuation in sequential depressive symptoms across a twelve-month period, the RMSSD of depressive symptom change was calculated. As previously stated, individuals’ composite PHQ-9 scores collected at months 0, 3, 6, 9, and 12 were used to calculate variability in depressive symptoms (RMSSD). Thus, an individual’s PHQ-9_*RMSSD*_ represented a single metric of depressive symptom variability that captured fluctuation in symptom expression across the entire study. Additionally, PHQ-9_*RMSSD*_ was correlated with mean PHQ-9 score to establish that PHQ-9_*RMSSD*_ was not simply a proxy for depression symptom intensity (*r* = 0.54, *R*^2^ = 0.29).

### Machine learning modeling approach

The present analyses were completed in Python (v 3.9) [47], and followed a threefold cross-validation framework (80%), allowing for a within-sample completely held-out test set (20%) to quantify predictive performance [48], and providing an efficacious approach in allowing for unbiased performance estimates in machine learning modeling [49]. Specifically, a stacked ensemble machine learning approach was used across the five MICE-generated datasets to assess for predictive robustness across the plausible imputation distribution. Stacked ensemble machine learning approaches have shown the capacity to consistently outperform base algorithms in detecting depression [50], by leveraging algorithmically distinct machine learning models (e.g., linear models, tree-based models) to individually train on the data. The individual model predictions are subsequently used as inputs to a final “meta” model, which returns a consensus score. The stacked ensemble algorithms and hyperparameters implemented for the present analysis are provided in Supplementary Table 2. In addition, the cross-validation architecture and random seed chosen for splitting the data were standardized across the three models (baseline biodemographic model; passively collected movement and sleep model; composite model) to reflect consistency across the model progression. Further, an exhaustive feature-inclusion approach was implemented, where all originally collected features were incorporated or transformed for the three respective model types (see Table 1) to evaluate performance with an increased feature space.

**Table 1:** (A) Model performance of the theory-organized and full variable set stacked ensemble machine learning approaches for the validation and held-out test set(s) for the three model types, reported as correlation ± standard deviation; (B) model performance of the theory-organized and full variable set stacked ensemble machine learning approaches for the validation and held-out test set(s) for the three model types, reported as normalized mean absolute error ± standard deviation.

(A)									
Modeling approach	Model 1: Demographic Information Model			Model 2: Movement and Sleep Information Model			Model 3: Composite Model		
	Variable number	*r* ± SD (test set)	*r* ± SD (validation set)	Variable number	*r* ± SD (test set)	*r* ± SD (validation set)	Variable number	*r* ± SD (test set)	*r* ± SD (validation set)
Theory-Organized Stacked Ensemble	8	0.27 ± 0.00	0.27 ± 0.03	24	0.27 ± 0.01	0.22 ± 0.08	32	0.33 ± 0.01	0.31 ± 0.04
Full Variable Set Stacked Ensemble	49	0.34 ± 0.00	0.35 ± 0.09	222	0.28 ± 0.01	0.25 ± 0.07	271	0.39 ± 0.01	0.35 ± 0.08

(B)									
Modeling approach	Model 1: Demographic Information Model			Model 2: Movement and Sleep Information Model			Model 3: Composite Model		
	Variable number	MAE_norm_ ± SD (test set)	MAE_norm_ ± SD (validation set)	Variable number	MAE_norm_ ± SD (test set)	MAE_norm_ ± SD (validation set)	Variable number	MAE_norm_ ± SD (test set)	MAE_norm_ ± SD (validation set)
Theory-Organized Stacked Ensemble	8	0.14 ± 0.00	0.11 ± 0.01	24	0.14 ± 0.00	0.11 ± 0.00	32	0.14 ± 0.00	0.11 ± 0.00
Full Variable Set Stacked Ensemble	49	0.13 ± 0.00	0.11 ± 0.00	222	0.14 ± 0.00	0.11 ± 0.00	271	0.13 ± 0.00	0.11 ± 0.00

### Model performance

Model performance was reported for the validation and held-out test set for each of the machine learning models as the mean and standard deviation across the five MICE-imputed datasets for correlative strength (*r*), and normalized mean absolute error (MAE_*norm*_). The MAE_*norm*_ reflects an outcome-agnostic representation of the model’s mean absolute error by dividing the mean absolute error by the range of the observed outcome, and thus represents the mean percentage error of the prediction.

### Model introspection

**Fig. 2:**
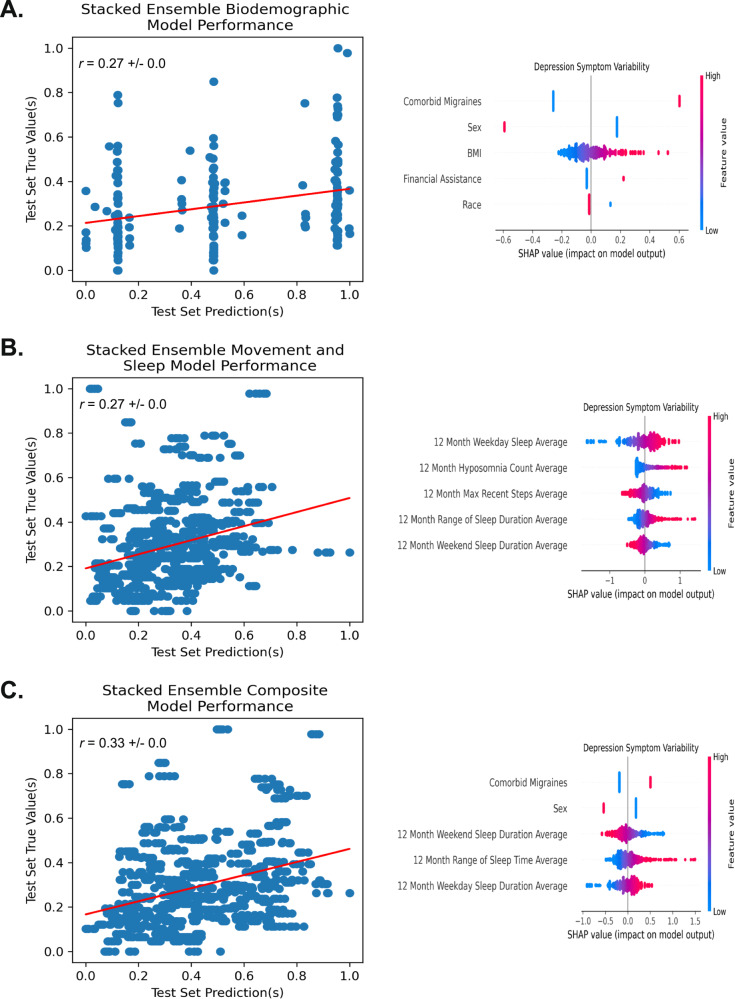
Model(s) actual versus predicted values plotted with respective correlative strength and the top five most influential features for the models’ predictions. In the respective SHAP plots, the individual dot color corresponds to the value of the variable, and location on the plot’s *x* axis corresponds to that point’s relative impact on the model output (e.g., a high-feature value (red) with a corresponding high *x* axis value (SHAP value) represents a point that strongly, positively influences the model’s prediction of depression symptom variability). **A** Baseline biodemographic variables. **B** Passively collected movement and sleep variables. **C** A composite model, using biodemographic and passively collected movement and sleep variables. *r* = Pearson’s correlation coefficient. For binary features, the presence of comorbid migraines, male sex, required financial assistance, and white race represented a higher feature value (red SHAP value color).

To assess the most influential features for model prediction across the three models, SHapley Additive exPlanations (SHAP) were implemented, and the top five most influential features were reported for each model. SHAP provides a method for model introspection by iteratively perturbing the input features and assessing how this affects the model prediction [51]. Thus, SHAP provides a mechanism for determining feature importance, as well as the marginal contribution of each input variable to the model’s prediction at the individual level, represented as the individual values positioning on the *x* axis of Fig. 2. Specifically, an individual features SHAP values can be interpreted as the features’ partial association with the outcome when controlling for all other input features in the model. Collectively, SHAP can estimate the relative magnitude of a feature’s influence on a model’s predictions, directional relationships between features and predicted outcomes, as well as different order interactions between features.

## Results

### Baseline biodemographic features

#### Baseline biodemographic modeling results

Baseline biodemographic features were incorporated into a stacked ensemble machine learning approach to detect depression symptom variability (PHQ-9_*RMSSD*_) (Supplementary Table 1). Averaged across the five MICE-imputed datasets, we found a weak, positive correlation (*r* = 0.27 ± 0.00, MAE_*norm*_ 0.14 ± 0.00; see Table 1) between predicted long-term depression symptom variability outcomes and actual long-term depression symptom variability outcomes in the held-out test set (see Fig. 2A).

#### Relative feature importance and directionality for the baseline biodemographic model

Using SHAP (see Methods section Model introspection), we found comorbid migraines to be the most influential feature in the model’s prediction of higher depression symptom variability, followed by female sex, high body mass index (BMI), required financial assistance, and non-White race (see Fig. 2A and Supplementary Table 1).

### Passively collected movement and sleep features

#### Passively collected movement and sleep modeling results

Statistically derived features from wearable, passively collected movement and sleep data (Supplementary Table 1) were incorporated into a stacked ensemble machine learning model to detect depression symptom variability (PHQ-9_*RMSSD*_). Similar to the biodemographic model, when averaged across the five MICE-imputed datasets, we found a weak, positive correlation (*r* = 0.27 ± 0.01, MAE_*norm*_ 0.14 ± 0.00; see Table 1) between predicted long-term depression symptom variability outcomes and actual long-term depression symptom variability outcomes in the held-out test set (see Fig. 2B).

#### Relative feature importance and directionality for the passively collected movement and sleep model

Using SHAP (see Methods section Model introspection), we found (1) high weekday sleep duration, (2) high count of nights with less than five hours asleep (hyposomnia) in the last week, (3) lower recent step count, (4) high range of sleep duration, and (5) low weekend sleep duration to be the top five most influential features in the model’s prediction of high depression symptom variability (see Fig. 2B and Supplementary Table 1). The top five features in the passively collected movement and sleep reflect an average over twelve months.

### Combined biodemographic and passively collected movement and sleep features

#### Biodemographic and passively collected movement and sleep modeling results

Using a composite model of baseline biodemographic features (see Results section Baseline biodemographic features) and statistically derived features from wearable passively collected movement and sleep data (see Results section Passively collected movement and sleep features) we found a moderate, positive correlation (*r* = 0.33 ± 0.01, MAE_*norm*_ 0.14 ± 0.00; see Table 1) between predicted depression score variability outcomes and actual depression score variability outcomes in the held-out test set (see Fig. 2C).

#### Relative feature importance for the combined biodemographic and passively collected movement and sleep model

Using SHAP (see Methods section Model introspection), we identified (1) comorbid migraines to be most influential in the model’s prediction of high depression symptom variability (PHQ-9_*RMSSD*_), followed by (2) female sex, (3) lower duration of weekend sleep, averaged over 12 months, (4) higher range of time asleep, averaged over 12 months. and (5) higher duration of weekday sleep, averaged over 12 months (see Fig. 2C and Supplementary Table 1).

### Exhaustive feature-inclusion modeling results

**Fig. 3:**
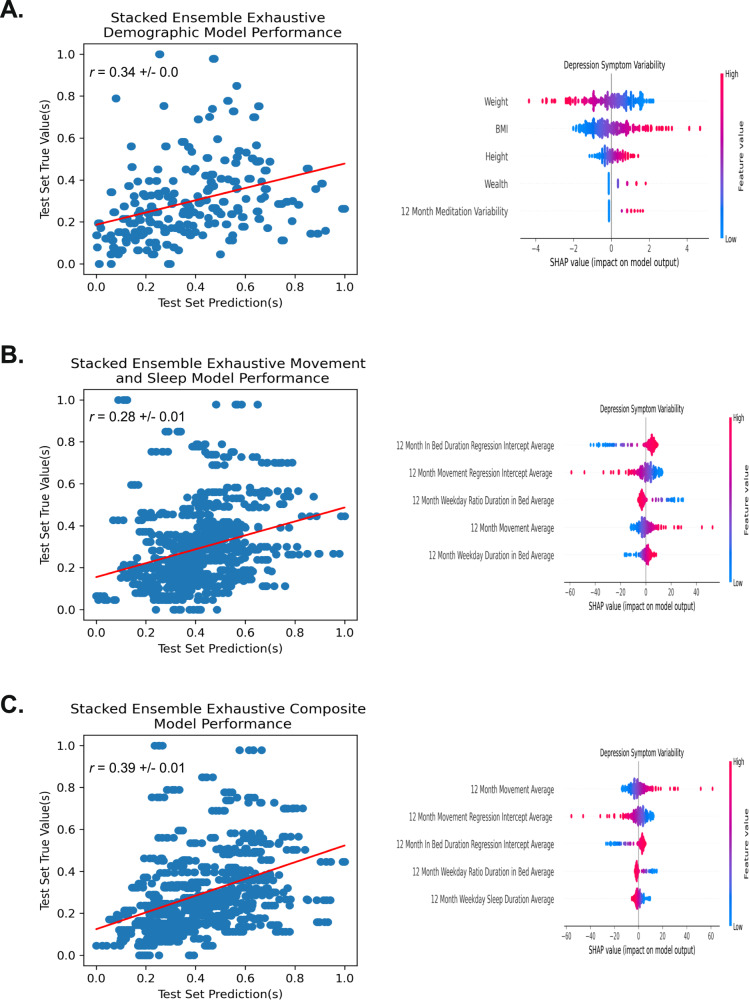
Comparative analysis incorporating or transforming all originally collected variables for the three respective models. Model(s) actual versus predicted values plotted with respective correlative strength and the top five most influential features for the models’ predictions. In the respective SHAP plots, the individual dot color corresponds to the value of the variable, and location on the plot’s *x* axis corresponds to that point’s relative impact on the model output (e.g., a high-feature value (red) with a corresponding high *x* axis value (SHAP value) represents a point that strongly, positively influences the model’s prediction of depression symptom variability). **A** Baseline biodemographic variables. **B** Passively collected movement and sleep variables. **C** A composite model, using biodemographic and passively collected movement and sleep variables. *r* = Pearson’s correlation coefficient.

Complementing the decision to subset biodemographic and passively collected movement and sleep features using theoretical and empirical domain knowledge, we also constructed three parallel stacked ensemble machine learning models operating on the non-subsetted PSYCHE-D [40] feature set, including 49 original and statistically derived biodemographic features, and 222 statistically derived movement and sleep features. The exhaustive feature-inclusion approach showed marginal performance improvement compared to the theory-driven variable selection approach across the three model types (see Table 1 and Fig. 3). Nevertheless, the exhaustive inclusion of all previously collected features introduced increased model complexity and reduced featured interpretability.

## Discussion

### General overview

The present results demonstrate the successful application of both biodemographic and passively collected movement and sleep features for modeling the novel outcome, long-term depression symptom variability. We found moderate predictive capacity of the biodemographic and passively collected movement and sleep features for long-term depression symptom variability detection when used in concert. This validates our hypothesis (1) of features indicative of depression severity also indicative of depression symptom variability and (2) the predictive utility of complementarity (i.e., unique information) between feature types. Regarding our theory-guided subsetting approach, we found modest improvements in predictive performance using a non-subset feature set with an increase in model complexity (see Table 1 and Fig. 3).

### Implications and importance

The successful application of the biodemographic and passively collected movement features used in the present analysis to detect depression symptom variability has promising mental health clinical implications, strengthening evidence for more objective and naturalistic assessments, with less burden to patients [52]. The work also validates our hypothesis of variables empirically correlated with major depressive disorder (e.g., sex, migraines, sleep disturbances) also having association with depression symptom variability. While biomarkers of depression severity have been studied more extensively, factors associated with depression symptom variability have had relatively less attention.

In this work, we make the case for (1) variability, per se, as an outcome of high importance, as well as (2) the importance and utility of predicting who is likely to have high variability. First, variability has been linked to important outcomes, including suicide attempts in high-risk individuals [13], as well as family functioning in the case of maternal depression [14]. Thus, symptoms variability, itself, may be a risk factor for important clinical outcomes. Second, long-term symptom variability is a necessary precondition for episodic depression relapse and remission. Relapse and remission counts have obvious importance as clinical outcomes by themselves, and have been associated with poorer long-term prognosis in MDD [53, 54]. Third, predicting person-level variability has implications for personalized medicine [55] approaches to mental healthcare. Identifying who is likely to have higher symptom variability over time, would allow for person-tailored assessment frequencies. For instance, a person with high depression symptom variability would require more frequent depression assessments compared to someone with lower depression symptom variability to adequately capture the disorder course over time.

### Model introspection and depression symptom variability theory

The presence of migraines was the most influential of the biodemographic features for predicting depression symptom variability and remained so even when combined with statistically derived passively collected movement and sleep features (see Fig. 2). Migraines have been established as highly comorbid with depression [56, 57]; additionally, research has demonstrated that migraines may perturb the naturalistic course of depression, prolonging the time to depression remission [58]. However, the direct relationship of migraines to depression symptom variability is not well understood. A plausible explanation stems from research demonstrating depression exacerbation in concurrence with migraine headache onset (a phenomenon reported in nearly one-third of a depressed sample) [59]. Given the discrete and episodic nature of migraine headaches [60], as well as the empirical support for simultaneity in migraine onset and depression exacerbation, it would follow that such patients would show heightened variability in their depression over time.

Following migraines, the next most influential features for modeling depression symptom variability in the biodemographic model included: (i) female sex, (ii) high BMI, (iii) required financial assistance, and (iv) non-White race. These findings may be contextualized in research to date, which demonstrated females had a considerably higher rate of depressive episodes [61], with higher frequency, theoretically serving as a proxy for variability. Further, required financial assistance may be a proxy for lower socioeconomic status, a known correlate of depression [62]; specific to variability, a large longitudinal cohort study (*N* = 12,650) showed socioeconomic status predicted long-term patterns of change in intra-individual depression symptom variability [63]. However, it is also important to consider that markers of variability in depression, such as race and sex, could also be markers for events such as racism and discrimination, which may, themselves, have an episodic course [64]. While racism and discrimination have been shown to predict depressive symptoms, longitudinally [65], discriminatory events have also been shown to cause acute exacerbations in depression [66]. Such depression “spikes” over time may appear to be of a more variable course.

Movement and sleep features derived from passively collected actigraphic data demonstrated capacity for modeling depression symptom variability. Sleep behaviors were highly represented among the most influential features in the movement and sleep model, as well as the composite model (see Fig. 2B, C). Specifically, sleep duration (for both weekends and weekdays), range of sleep duration, and nights spent with hyposomnia were the most influential sleep-related features. These findings are generally consistent with well-established knowledge of the close relationship between sleep, activity, and depression [6, 67], validated with passively collected, objective data [33]. Notably, sleep quality and duration have bidirectional associations with psychosocial functioning amongst young adults [68]. Moreover, short sleep duration and poor sleep quality are associated with a higher prevalence of depressive symptoms among university students [69]. This suggests a complex relationship between sleep and depression that is not merely unidirectional, but rather complicated by biopsychosocial variables.

Further, specific sleep profiles have been empirically correlated with longitudinal depression symptom variability [70], perhaps suggesting the existence of sleep markers for MDD variation. Curiously, sleep quality correlates more strongly with psychosocial functioning than sleep duration among young adults [68]. Our findings, range of sleep duration, nights with hyposomnia, and sleep duration, may be further contextualized in research linking similar features (i.e., total sleep time and day-to-day variability in total sleep time) to next-day mood and depressive symptoms [71]. It follows that changes in mood may track with changes in sleep; thus, a higher range of nightly sleep duration would imply a wider range of depression severity. Recognizing the multifactorial nature of sleep, optimizing sleep architecture, quality, and duration collectively, yet intricately, influences depression outcomes. Both insufficient and excessive sleep durations have been shown to elevate depression risk [72, 73], with the latter being particularly pertinent when coupled with sustained poor sleep quality. Factors such as emotional exhaustion and stress, whether stemming from academic demands [74] or shift work [75], further complicate the intricate relationship between sleep and depression.

Recall that, in addition to a feature subsetting approach, guided by a priori domain knowledge, we comparatively tested an exhaustive feature set approach, using all biodemographic and all movement and sleep features (see Fig. 3C). Despite the reduced interpretability of such a model, conferred by the inclusion of statistical features which are more convoluted, there is a modest increase in performance (*r* = 0.39, compared to *r* = 0.33 with reduced feature model), highlighting the utility and application of such an approach for a performance-driven task. In contrast to the domain-driven approach, the top five most influential features in the exhaustive feature model were all derived from passively collected movement and sleep data—none from biodemographic information or self-report. Notably, a subset of these features were generated from regression-based statistics on the passively collected movement and sleep data [35], which have not been established in the literature on long-term depression symptom variability, but do seem to offer a substantive increase in information for the model’s predictions, allowing for increased model performance. These findings suggest further consideration into the utility of feature engineering as it pertains to passively collected movement and sleep data, as it offers clear advantages for tasks strictly concerned with improving predictive performance relating to long-term depression symptom variability.

### Strengths, limitations, and future directions

The current study uniquely utilized long-term depression variability as an outcome measure. In addition, our methods allow for a direct comparison between feature selection strategies, specifically theory-informed versus exhaustive, and between feature types, specifically passive sensing-derived features and baseline demographic features. A significant strength of our work lies in our application of a robust stacked ensemble approach, accommodating the potentially complex relationships among features. Despite the strengths and novelty of our work, the study results must be considered in the context of several important limitations, described here. (1) The study population was limited in demographic diversity, and future research would benefit from analyzing a more nationally representative sample when detecting depression symptom variability. Further, a consideration for depression symptom variability within demographic groups (e.g., gender, race) should be assessed, as influential biodemographic and passively collected movement and sleep features are likely differentially expressed between populations, which would allow for more effective personalized treatment. (2) Recall that the outcome (*PHQ-9*_*RMSSD*_) is derived from self-reported PHQ-9 scores at 3-month intervals over the course of one year. As such, the temporal resolution of depression symptom variability is limited. A related but distinct limitation inherent in the original study design is the mismatch between the 2-week look-back period of the PHQ-9 and the 3-month interval at which the measurements were collected. In future research investigating depression symptom variability, ecological momentary assessments for depressive symptoms would be preferable. (4) Finally, the choice of one year over which to measure variability has important implications in the applicability and interpretation of results. While one year is likely sufficient to capture a single depressive episode [76], it may be insufficient to capture the temporal dynamics across multiple depressive episodes. Furthermore, while the present investigation of factors associated with depression symptom variability is appropriately conducted on a community sample, given that over one-third of participants (38.8%) reported PHQ-9 scores both below and above the clinical threshold for depression (PHQ-9 ≥ 10), generalizability to a clinical sample remains uncertain. Thus, a future extension of this work would be validation and comparison on a clinical sample to assess both model performance as well as features most associated with the model’s predictions.

## Conclusion

In the present work, we emphasize depression symptom variability as an important clinical and research variable in mental health. Variability represents an important attribute of the depression’s longitudinal course, as well as a dimension of heterogeneity between depressed persons. In addition, depression symptom variability has been linked to important clinical outcomes, such as suicide. Though much is known of factors associated with point-in-time depression severity, relatively little is known of long-term, naturalistic variability in depression, as well as person-specific factors which associate with variability. In the present work, we explore the capacity of biodemographic and passively collected movement and sleep information to model depression symptom variability. We find positive results to suggest association between both biodemographic and passively collected data types, independently, as well as evidence of complementarity in predictive capacity. Our work provides an early step toward the complementary, personalized use of unobtrusive data types in addressing the question of depression’s temporal variability.

### Supplementary information

Supplementary Material

## Data Availability

The Prediction of Severity-Change Depression (PSYCHE-D) dataset used in the present manuscript can be accessed at https://zenodo.org/records/5085146.

## Code availability

The data that support the findings of this study are available from the corresponding author, GP, upon reasonable request.
