# Supplementary material for: Using digital phenotyping to capture depression symptom variability: detecting naturalistic variability in depression symptoms across one year using passively collected wearable movement and sleep data
Source: Transl Psychiatry. 2023 Dec 9;13:381. doi: 10.1038/s41398-023-02669-y (PMC10710399; doi:10.1038/s41398-023-02669-y)
Supplement: Supplementary file 1 — Supplementary Material [file 41398_2023_2669_MOESM1_ESM.docx]

**Supplementary Table 1**

| **Variable Name** | | **Variable Description** |
| --- | --- | --- |
|  |  | *Biodemographic* |
| Sex |  | Female/Male [(Hasin et al., 2018; Maier et al., 1999)](https://www.zotero.org/google-docs/?k5yrq6). |
| Race |  | White/Non-White [(Riolo et al., 2005)](https://www.zotero.org/google-docs/?3i9ZOU). |
| Body Mass Index (BMI) |  | Continuous height and weight measurement [(de Wit et al., 2009)](https://www.zotero.org/google-docs/?xpwHDj). |
| Pregnant |  | Pregnant/Not Pregnant [(Amiel Castro et al., 2017; Ryan et al., 2005)](https://www.zotero.org/google-docs/?eVpNt5). |
| Money Assistance |  | Money assistance from the government in the last month [(Everson et al., 2002)](https://www.zotero.org/google-docs/?p9zVFY). |
| Comorbid Diabetes, Type 1 |  | Diagnosed Type 1 Diabetes: Yes/No [(Anderson et al., 2001; Roy & Lloyd, 2012)](https://www.zotero.org/google-docs/?qyiTAR). |
| Comorbid Diabetes, Type 2 |  | Diagnosed Type 2 Diabetes: Yes/No [(Anderson et al., 2001; Roy & Lloyd, 2012)](https://www.zotero.org/google-docs/?2eYKx6). |
| Comorbid Migraines |  | Diagnosed Migraines: Yes/No [(Jahangir et al., 2020; Molgat & Patten, 2005)](https://www.zotero.org/google-docs/?OTW3zg). |
|  |  | *Movement* |
| Mean awake activity |  | The average number of steps taken while awake while wearing the device [(Fekedulegn et al., 2020)](https://www.zotero.org/google-docs/?nuovEO). |
| Time spent performing low and moderate-to-vigorous physical activity |  | IQR of number of minutes with low and moderate-vigorous physical activity range steps [(Alexandre Domingues et al., 2014; Tudor-Locke et al., 2011)](https://www.zotero.org/google-docs/?DJLRxx). |
| Days of active versus sedentary movement across the week |  | Number of days with more 10,000 or less than 5,000 steps in the last 7 days [(Alexandre Domingues et al., 2014; Tudor-Locke et al., 2011)](https://www.zotero.org/google-docs/?ink6Cq). |
|  |  | *Sleep* |
| Nighttime sleep variability |  | Interquartile range of minutes sleeping per night [(Patapoff et al., 2022)](https://www.zotero.org/google-docs/?ZeenTG). |
| Mean nighttime sleep on weekdays |  | Mean number of minutes sleeping per night on weekdays [(Du et al., 2022)](https://www.zotero.org/google-docs/?yCZmjo). |
| Mean nighttime sleep on weekends |  | Mean number of minutes sleeping per night on weekends [(Du et al., 2022)](https://www.zotero.org/google-docs/?Bx5ixd). |
| Sleep start time |  | Median start hour of main sleep [(Du et al., 2022)](https://www.zotero.org/google-docs/?uzsdKU). |
| Variability in sleep start time |  | Interquartile range of start hour of main sleep [(Patapoff et al., 2022)](https://www.zotero.org/google-docs/?TErNSF). |
| Weekly hypersomnia count |  | Number of nights with >10 hours asleep, last 7 days [(Dauvilliers et al., 2013; Plante, 2022)](https://www.zotero.org/google-docs/?G1z9Of). |
| Weekly hyposomnia count |  | Number of nights with <5 hours asleep, last 7 days [(Institute of Medicine (US) Committee on Sleep Medicine and Research, 2006; Plante, 2022)](https://www.zotero.org/google-docs/?8FQUXR). |

*Supplementary Table 1. The independent variables used in the stacked ensemble machine learning model reflecting: variable name, description, and previously documented associations of the variable, or related information, with depression-related constructs.*

**Supplementary Table 2**

*Machine Learning Model Architecture and Hyperparameters*

| *Modeling*  *Approach* | *Upper-Level Machine Learning Algorithm*  *[Hyperparameters]* | *Lower-Level Machine Learning Algorithm(s) [Hyperparameters]* |
| --- | --- | --- |
| *Stacked Ensemble* | *Ridge Regressor [alpha = 0.1]* | *Support Vector Regressor* [kernel = linear]  *Linear Regressor*  *XGBoost Regressor* [booster = gblinear,  learning rate = 0.0001] |

*Supplementary Table 2. Stacked ensemble model algorithms and hyperparameters used for detecting long-term depression variability.*
